# pH-Selective Cytotoxicity of pHLIP-Antimicrobial Peptide Conjugates

**DOI:** 10.1038/srep28465

**Published:** 2016-06-23

**Authors:** Kelly E. Burns, Tanner P. McCleerey, Damien Thévenin

**Affiliations:** 1 Department of Chemistry, Lehigh University, 06 East Packer Ave, Bethlehem, PA 18015, USA.

## Abstract

Positively charged antimicrobial peptides have become promising agents for the treatment of cancer by inducing apoptosis though their preferential binding and disruption of negatively charged membranes, such as the mitochondrial membrane. (KLAKLAK)_2_ is such a peptide but due to its polarity, it cannot cross the cellular membrane and therefore relies on the use of a delivery agent. For targeted delivery, previous studies have relied on cell penetrating peptides, nanoparticles or specific biomarkers. Herein, we investigated the first use of pHLIP to selectively target and directly translocate (KLAKLAK)_2_ into the cytoplasm of breast cancer cells, based on the acidic tumor micro-environment. With the goal of identifying a lead conjugate with optimized selective cytotoxicity towards cancer cells, we analyzed a family of (KLAKLAK)_2_ analogs with varying size, polarity and charge. We present a highly efficacious pHLIP conjugate that selectively induces concentration- and pH-dependent toxicity in breast cancer cells.

In search for novel and more efficacious anticancer therapeutics, many positively charged antimicrobial peptides (AMPs) have surfaced as promising agents because they can kill cancer cells by preferentially disrupting mitochondrial membranes while sparing plasma membranes. Indeed, while mitochondrial membranes maintain large transmembrane potentials and have a high content of anionic phospholipids, the outer leaflet of eukaryotic plasma membrane is almost exclusively composed of zwitterionic phospholipids and has a low membrane potential^1^,^2^,^3^,^4^,^5^. Thus, upon cytoplasmic delivery, an AMP would induce cell death by preferential mitochondrial membrane disruption, while remaining non-toxic outside the cells^5^. Therapeutic strategies that induce mitochondrial depolarization are attractive because they bypass apoptosis resistance mechanisms that act upstream of the mitochondria^6^,^7^.

(KLAKLAK)_2_ is a cationic AMP that forms an amphipathic α-helix when bound to negatively charged lipid membranes^8^,^9^,^10^ and that can induce cell death in a variety of cell lines upon cell internalization^10^,^11^,^12^,^13^,^14^,^15^. Even though it has been shown to be able to accumulate on, disrupt and depolarize the mitochondrial membrane, contrasting mechanisms of cell death have been proposed for (KLAKLAK)_2_-based cancer therapeutics. Although some reports indicate that these peptides induce apoptosis due to their ability to depolarize mitochondrial membranes^10^,^11^,^12^,^13^,^14^, others have reported that they induce plasma membrane lysis leading to oncotic/necrotic death in cancer cells in caspase-dependent and caspase-independent pathways^14^,^15^. This discrepancy in mechanisms of action is very likely due to the difference in the methods used to target and translocate (KLAKLAK)_2_ conjugates to and into cancer cells^10^,^11^,^12^,^13^,^14^,^15^. Indeed, the efficacy of (KLAKLAK)_2_ is challenged by its poor ability to permeate the plasma membrane^10^, and it therefore requires the use of a delivery agent to facilitate its cellular uptake. Several methods have been implemented for the delivery of (KLAKLAK)_2_ including, membrane receptor ligands, antibodies, cell penetrating peptides^12^, and nanoparticles^16^. However, these strategies either are effective at rather high concentration (~100 μM)^10^, lack specificity for cancer cells (e.g., cell penetrating peptides), or rely on the over-expression of biomarkers on the surface of cancer cells to achieve their targeting. While over-expression provides a window of selective targeting, uptake into normal tissues is seen and has the potential to lead to unacceptable toxicity profiles. Indeed, preclinical and clinical evidence demonstrates that therapy strategies based on the targeting of specific proteins is significantly hampered by tumor heterogeneity, which can promote tumor evolution, leading to the loss of cell surface proteins and, eventually, to therapy resistance and disease progression. Consequently, relying on a general feature of tumors as the basis for developing a targeted therapy may be more beneficial than relying a single cancer biomarker. Moreover, it is not clear how lytic peptides internalized through endocytosis and how they escape the endosome to reach the mitochondrial membrane. Thus, there is a need for a delivery agent capable of enhancing the selectivity, potency and spectrum of the malignant cells that can be targeted by AMPs and (KLAKLAK)_2_, specifically.

In the present study, with the goal of inducing selective toxicity in cancer cells, we employ the tumor-targeting peptide pH(Low) Insertion Peptide (pHLIP) for the specific targeting and delivery of a family of (KLAKLAK)_2_ derivatives directly into the cytoplasm. The targeting achievable by pHLIP is based on the acidic tumor micro-environment that is exhibited by most solid tumors, regardless of their tissue or cellular origin. At a pH corresponding to the extracellular pH of tumors, pHLIP undergoes a pH-dependent conformational change driven by the protonation of aspartic acid residues, promoting the insertion of its C-terminus across the cell membrane to form a transmembrane α-helix^17^,^18^. Remarkably, unlike other drug delivery system strategies, pHLIP alone can target tumors, carry cargo and translocate the payload across the plasma membrane without relying on cell receptor interactions or by the formation of pores within the membrane^19^,^20^. pHLIP has been shown to translocate a plethora of cargo molecules across the plasma membrane into the cytoplasm of cancer cells^21^,^22^,^23^,^24^,^25^,^26^, and to target solid tumors as well as metastasis foci in mice^26^,^27^,^28^,^29^. We have also recently shown that pHLIP can target the potent antimitotic monomethyl auristatin E to breast cancer xenographs in mice^23^.

Here, we show that (KLAKLAK)_2_ derivatives, when conjugated to the C-terminus of pHLIP, induce a pH-selective toxicity effect in breast cancer cells from mitochondrial membrane disruption (Fig. 1). This study is the first not only to employ pHLIP to deliver antimicrobial peptides into cancer cells but also to test the size, polarity and charge of the cargo peptide systematically. We thus anticipate that this approach could be applied to a broad range of therapeutic peptides with intracellular targets.

## Results and Discussion

**Figure 1:**
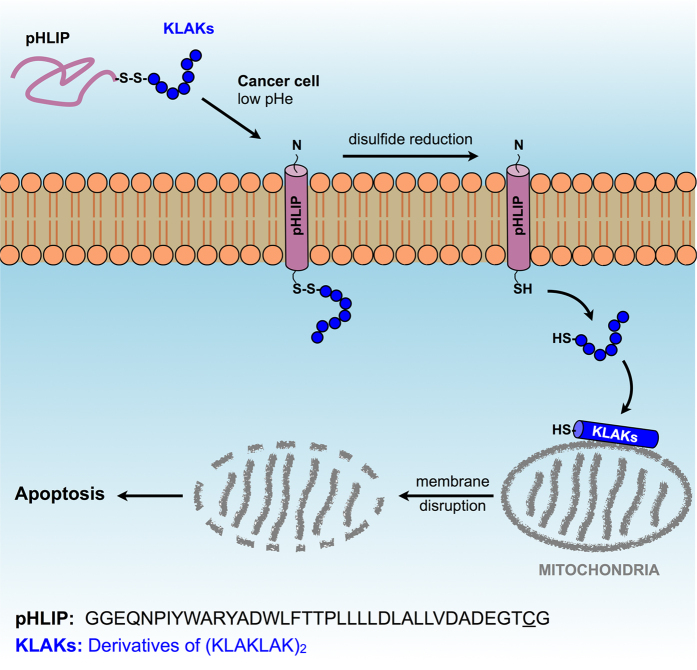
Schematic representation of the hypothesized effect of the pHLIP–KLAKs on apoptosis and cell viability. The peptide sequences of pHLIP is shown.

While (KLAKLAK)_2_ has been shown to be efficacious at inducing cell death when transported into cells, its relatively large size and high polarity were a concern, as the properties of cell-impermeable cargo molecules that can be delivered into cells by pHLIP are not fully defined. Indeed, even though pHLIP has been shown previously to be capable of translocating across membrane highly polar peptides (octanol/water partition coefficient [Log P_o/w_] ~ 3) in a pH-dependent manner, these peptides were cyclic and substantially smaller than (KLAKLAK)_2_ (molecular weight ~800 g.mol^−1^)^21^. Thus, with the goal of optimizing delivery by pHLIP and toxicity towards cancer cells, we tested, in addition to (KLAKLAK)_2_, smaller peptide analogs ranging in size and polarity (Table 1). The four-amino acid peptide analogues, KAAK, KKKK, KLLK and KGGK have been shown previously to exhibit, when covalently linked to a fatty acid (lipopeptide), potent broad-spectrum antimicrobial activity against a range of fungi, Gram-positive and Gram-negative bacteria, albeit with varying specificity^30^,^31^,^32^. We also tested the activity of the 4-amino acid KLAK as well as of the 7-amino acid KLAKLAK peptide because its size and polarity make it an attractive compromise to the long and polar (KLAKLAK)_2_ and the short four-amino acid peptides. Importantly, these six shorter analogues have never been tested for their activity against mitochondrial membranes upon intracellular delivery.

(KLAKLAK)_2_ and its six peptide derivatives (subsequently designated KLAKs) were synthesized and conjugated to the C-terminus of pHLIP through a disulfide bond, allowing for the antimicrobial peptides to act on the mitochondrial membranes upon cytoplasmic entry and disulfide reduction (Fig. 1). KLAKs, pHLIP and conjugates were purified by RP-HPLC and identified by MALDI-TOF MS (See Experimental Section).

The efficacy of the pHLIP-KLAKs constructs to inhibit cell proliferation in a concentration- and pH-dependent manner was investigated in MDA-MB-231 breast cancer cells. Cell treatments were conducted from 10 μM down to 2.5 nM of pHLIP-KLAKs at either pH 7.4 or 5.0 for 2 hours at 37 °C, after which constructs were removed and cells were recovered for 72 hours at normal pH in complete media before assessing cell viability using the MTT assay. Noteworthy, this treatment protocol, which was chosen to prevent any unwanted effects to the cells due to the prolonged exposure to a low pH environment, is much shorter and stringent than the 72-hour treatments typically used with (KLAKLAK)_2_^10^,^14^,^15^,^33^. We have previously shown that this treatment protocol and pHLIP alone have no effect on MDA-MB-231 cell viability^23^,^24^.

**Figure 2:**
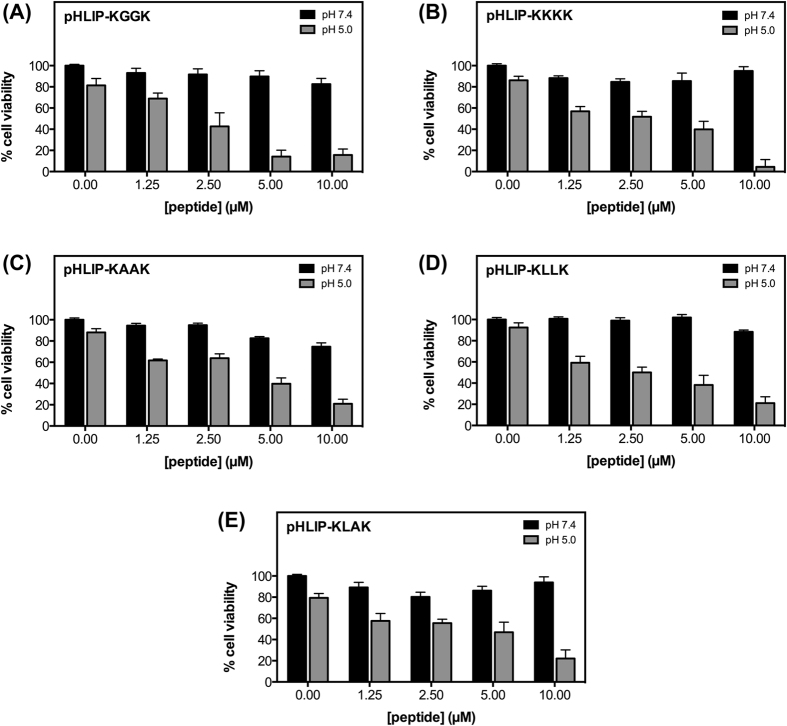
Inhibition of cell growth. MDA-MB-231 cells treated with 4-amino acid pHLIP-KLAKS conjugates. Black columns represent cells treated at pH 7.4 and cells treated at low pH are shown in grey. For each condition, 3,000 cells/well (96-well plate) were seeded, allowed to adhere overnight, treated for 2 hours, washed once with media and cultured for 72 hour in complete medium at physiologic pH. Cell viability was assessed with the MTT assay. All measurements were normalized to the media control (0 μM, pH 7.4), as 100% cell viability. Results are shown as mean ± SEM (n = 9–12).

Treatments with the tetrapeptide pHLIP-KLAKs conjugates were carried out from 1.25 μM to 10 μM. As hypothesized, little to no effect on MDA-MB-231 cell viability was observed when cells were treated with these constructs at normal physiological pH (Fig. 2, black bars). On the other hand, cell proliferation was drastically disrupted when cells were treated at low pH (up to 95% growth inhibition) (Fig. 2, grey bars). The anti-proliferative effect is also concentration-dependent, with cell viability ranging from 69% to 5%, with increasing treatment concentration ranging from 1.25 to 10 μM, respectively. Consistent with the fact that the palmitoyl-KGGK lipopeptide had the highest relative antimicrobial activity of all KXXK peptides when tested by Makovitzki *et al*. against bacteria, fungi and yeast^30^, pHLIP-KGGK showed the most overall cell proliferation inhibition of all the 4-amino acid pHLIP-KLAKs conjugates (Fig. 2A). Indeed, while pHLIP-KKKK, pHLIP-KAAK and pHLIP-KLLK showed also high proliferation inhibition at 10 μM (from 80% to 95%), their overall activity is lower than pHLIP-KGGK (Fig. 2B–D). The pHLIP-KLAK construct exhibited similar activity to that of pHLIP-KLLK with growth inhibition up to 80% with 10 μM treatments (Fig. 2E). Noteworthy, the anti-proliferative effect observed is not due to any intrinsic peptide cytotoxicity or to cell membrane disruption, as neither pHLIP(WT) nor the tetrapeptides has any significant harmful effects on the cell viability or plasma membrane integrity at 10 μM (Figure S1).

**Figure 3:**
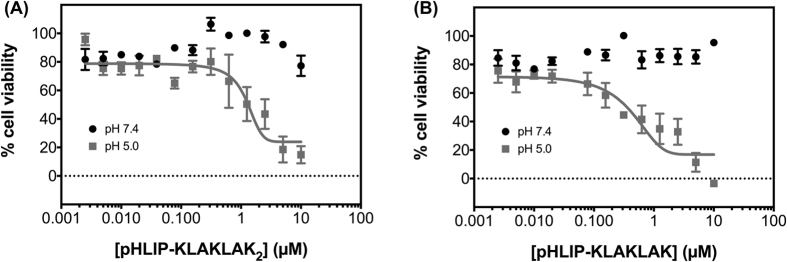
MDA-MB-231 cells treated with pHLIP-(KLAKLAK)_2_ (**A**), pHLIP-KLAKLAK (**B**), Black circles represent cells treated at pH 7.4 and cells treated at low pH are shown in grey. For each condition, 3,000 cells/well (96-well plate) were seeded, allowed to adhere overnight, treated for 2 hours, washed once with media and cultured for 72 hour in complete medium at physiologic pH. Cell viability was assessed with the MTT assay. All measurements were normalized to the media control (0 μM, pH 7.4), as 100% cell viability. Results are shown as mean ± SEM (n = 9–12). Data at pH 5.0 (A and B) were fitted with a sigmoidal dose-response using Prism 6 for Macintosh (GraphPad, Inc).

When cells were treated with pHLIP-(KLAKLAK)_2_ or pHLIP-KLAKLAK, concentration- and pH-dependent cell growth inhibitions were also observed, with little to no significant decrease in cell viability at pH 7.4 and up to 85% and 90% inhibition with pHLIP-(KLAKLAK)_2_ and pHLIP-KLAKLAK treatments, respectively (Fig. 3A,B). However, these pHLIP-(KLAKLAK)_2_ and pHLIP-KLAKLAK are significantly more cytotoxic than the 4-amino acid pHLIP-KLAKs conjugates, with IC_50_ values of 1 μM and 0.5 μM for pHLIP-(KLAKLAK)_2_ and pHLIP-KLAKLAK, respectively. We thus subsequently focused our efforts on these two conjugates.

It is important to point out that, while using short treatment time, the pHLIP-(KLAKLAK)_2_ and pHLIP-KLAKLAK constructs achieve greater cell growth inhibition at much lower concentrations when compared to most reported delivery methods. For instance, (KLAKLAK)_2_, when conjugated to the cyclic tumor-homing peptide CNGRC, achieved ~75% cell proliferation inhibition after 96 hours at 60 μM^10^. In another study, 30 μM of (KLAKLAK)_2_ conjugated to a monoclonal antibody targeting the prostate-specific membrane antigen were needed to induce 75% cell growth inhibition against LnCaP prostate cancer cells^14^. Finally, another approach, which relies on the formation of peptide amphiphile nanofibers that can be internalized by cancer cells, achieved IC_50_ values of ~6 μM in MDA-MB-231 and SKBR-3 breast cancer cell lines, though after 24 hour treatment times^15^. Noticeably, Sioud *et al*. showed that (KLAKLAK)_2_ when conjugated to a cancer-cell binding peptides induces rapid killing in breast cancer cells (10–20% cell viability in 1 h treatment), albeit through a very different apparent mechanism (i.e., disruption of the cytoplasmic membrane)^33^.

Although some reports indicate that (KLAKLAK)_2_ induces apoptosis due to its ability to depolarize mitochondrial membranes^10^,^14^,^15^,^34^, others have reported that it induces plasma membrane lysis leading to necrotic death in cancer cells^33^. We therefore investigated the possible mechanism(s) of cell death induced by the pHLIP-(KLAKLAK)_2_ and pHLIP-KLAKLAK constructs.

**Figure 4:**
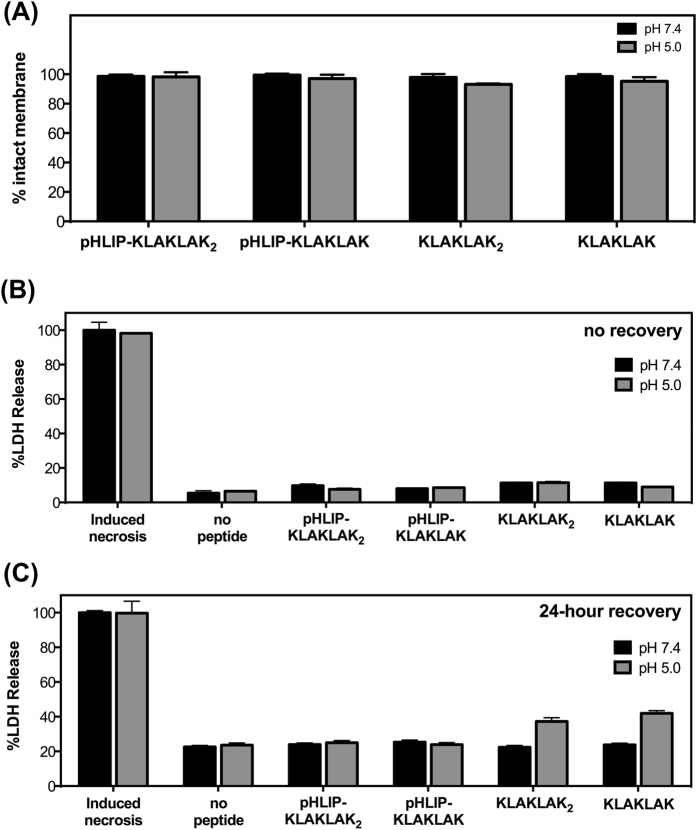
Effect of free peptides and pHLIP-conjugates on Plasma Membrane Integrity and Lactate Dehydrogenase (LDH) Release. (**A**) The integrity of the plasma membrane is assessed by the uptake of trypan blue dye. 3,000 cells/well of MDA-MB-231 cells were seeded, incubated until confluent (~72 hours) and treated with 10 μM for 2 hours at pH 7.4 (black bars) or pH 5.0 (grey bars). Cells were detached and counted based on trypan blue uptake with an hemacytometer: % of intact cells corresponds to the number of cells not showing any dye uptake over the total number of cells. Results are shown as mean ± SD (n = 3). (**B**) LDH release assay. Cells were seeded at a density of 200,000 cells/well in a 24 well plate, allowed to adhere overnight and treated with 10 μM for 2 hours at pH 7.4 (black bars) or pH 5.0 (grey bars). Cell media supernatant was monitored for the presence of LDH following treatment. Results are shown as mean ± SD (n = 3).

First, we assessed the effect of the conjugates on the integrity of cell membranes based on the uptake of Trypan Blue, which can be taken up by cells only if their plasma membrane is disrupted. Figure 4A shows that neither the conjugates nor free peptides causes disruption of cell membranes. We also measured the release of the intracellular enzyme lactate dehydrogenase (LDH) after peptide treatment, as another method to evaluate plasma membrane damage^35^. The results presented in Fig. 4B,C show that no significant LDH release is observed 2 and 24 hours after treatment. Importantly, it was previously shown that neither pHLIP alone nor low pH treatments disrupts membranes, including that of MDA-MB-231 cells^20^,^23^ (Figure S1). Thus, these results indicate that pHLIP-(KLAKLAK)_2_ and pHLIP-KLAKLAK do not cause cell death through dramatic disruption of the plasma membrane.

However, it is interesting to note that some LDH release is observed 24 hours post-treatment for cells treated with (KLAKLAK)_2_ or KLAKLAK at low pH (Fig. 4C), indicating that these two peptides have a tendency to disrupt the plasma membrane at lower pH. Therefore, while pHLIP-mediated translocation of cargo molecules is not thought to be mediated by endocytosis or passive diffusion through the membrane, we cannot exclude the possibility that the observed cytotoxicity of pHLIP-(KLAKLAK)_2_ and pHLIP-KLAKLAK might be associated to enhanced membrane permeability and/or membrane-disruptive ability of (KLAKLAK)_2_ and KLAKLAK at low pH. Indeed, acidification and subsequent formation of a pH-gradient across lipid membranes have been shown to facilitate membrane translocation of cationic cell penetrating peptides such as penetratin^36^,^37^. We thus also assessed the cytotoxicity of free (KLAKLAK)_2_ and KLAKLAK peptides against MDA-MB-231 cells.

**Figure 5:**
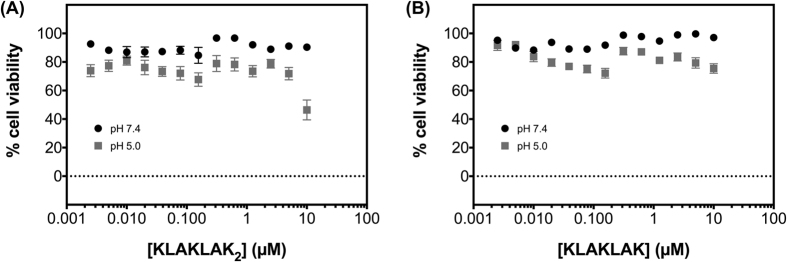
Effect of free peptides on cell viability. MDA-MB-231 cells were treated with 10 μM of either (KLAKLAK)_2_ (**A**) or KLAKLAK (**B**) at pH 7.4 (black circles) or pH 5.0 (grey squares) for 2 hours, washed once with media and cultured for 72 hours in complete medium at physiologic pH. Cell viability was assessed with the MTT assay, and all measurements were normalized to the media control (0 μM, pH 7.4), as 100% cell viability. Results are shown as mean ± SEM (n = 9).

Figure 5 shows that while (KLAKLAK)_2_ and KLAKLAK are not significantly toxic at physiological pH, consistent with previous observations in MDA-MB-231 cells^15^,^33^, both peptides exhibit enhanced cytotoxicity at low pH when used at 10 μM, albeit at different levels. (KLAKLAK)_2_ shows the greatest enhancement and KLAKLAK shows only a modest one, consistent with the number of ionizable groups and the enhanced permeability of such peptides at low pH. However, it is important to note that the pH-dependent cytotoxicity observed with pHLIP-(KLAKLAK)_2_ and pHLIP-KLAKLAK (Fig. 3) is unlikely due to free (KLAKLAK)_2_ and KLAKLAK, as no degradation products are observed by HPLC or mass spectroscopy after low pH treatment with pHLIP-(KLAKLAK)_2_ and pHLIP-KLAKLAK (Figure S2). Nevertheless, the significant killing of free (KLAKLAK)_2_ is an obvious concern in moving this conjugate forward, and we thus focused our subsequent effort solely on the pHLIP-KLAKLAK conjugate.

**Figure 6:**
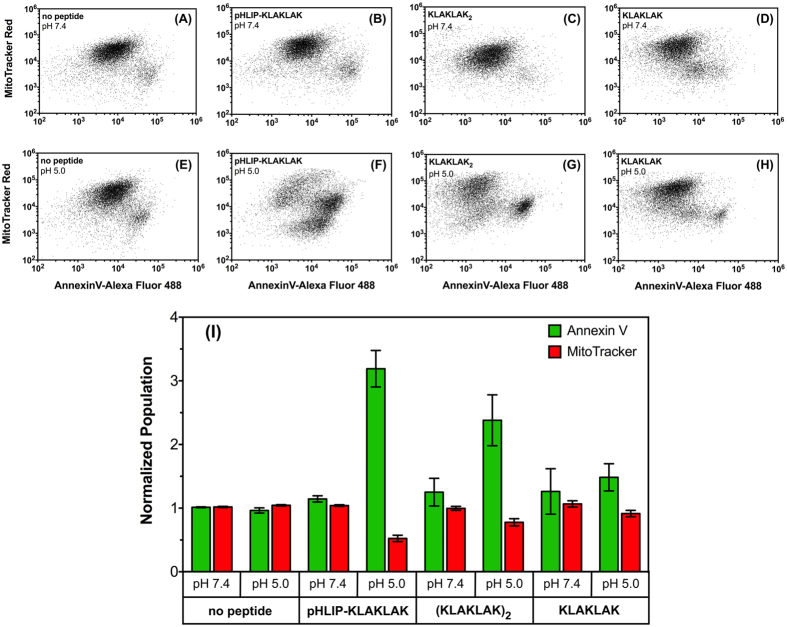
Induction of Apoptosis Due to Mitochondrial Membrane Disruption. Annexin V and MitoTracker Red dual staining was performed and measured by flow cytometry in MDA-MB-231 cells by pHLIP-KLAKLAK, (KLAKLAK)_2_ and KLAKLAK. (**A**–**H**) Cells were treated with either no peptide or with 10 μM peptides at pH 7.4 (**A**–**D**) or at pH 5.0 (**E**–**H**) for 2 hours, washed once with media and cultured for 24 hours in complete medium at physiologic pH. Quantification of mitochondrial depolarization and phosphatidyl serine externalization for cell samples was determined based on their fluorescence intensities at both 530 (Annexin V Alex Fluor 488) and 633 (MItoTracker Red) nm. Cells were gated as two populations based on high MitoTracker/low Annexin V and low MitoTracker/high Annexin V. (**I**) For all measurements the percentage of cells in each population was normalized to the media control (0 μM, pH 7.4). A minimum of 10,000 events were counted for each data point. The data was analyzed using the FACSDiva version 6.1.1 software. Results are shown as mean ± SD (n = 3).

To ensure that pHLIP-KLAKLAK exerts its activity by disrupting the mitochondrial membrane, we determined the extent of phosphatidylserine (PS) externalization and change in mitochondrial membrane potential, two of the hallmarks of (KLAKLAK)_2_ mechanism of action. These processes can be monitored by flow cytometry using Alexa Fluor 488-labeled Annexin V, which binds to cells based on their membrane composition and the MitoTracker Red that only binds to intact mitochondrial membrane^14^,^38^. Thus, live cells with intact mitochondrial membrane and low PS externalization should exhibit high MitoTracker Red and low Annexin V staining. This is the case, for example, with non-treated cells at pH 7.4 (Fig. 6A,I, Bar Set #1). On the other hand, cells ongoing cell death would exhibit increased green fluorescence (increase of PS externalization) and decreased red fluorescence (disruption of mitochondrial membrane) when compared to live cells. A clear shift in cell population should then be observed by flow cytometry. This is case, for example, when cells are treated with staurosporine, a known inducer of apoptosis (Figure S4).

As hypothesized and consistent with the results from the anti-proliferation assay (Fig. 3B), little to no effect on apoptosis markers was observed when MDA-MB-231 cells were treated with pHLIP-KLAKLAK at normal physiological pH (Fig. 6B,I, Bar Set #3). On the other hand, a significant increase in Annexin V signal and decrease in MitroTracker Red signal are observed when cells were treated with pHLIP-KLAKLAK at low pH, resulting in clear shift in cell population (Fig. 6F,I, Bar Set #4). Increase in Annexin V signal and decrease in MitroTracker Red signals are also indicative of the apoptotic process, which is often associated to the activation of caspases. Thus, to probe the possible involvement of a caspase-mediated apoptotic pathway, we monitored the activity of the apoptotic effector caspases 3 and 7 after treatment. Remarkably, our results (Figure S5) show that neither treatments with pHLIP-KLAKLAK or KLAKLAK induces any significant caspase activation. These results may appear contradictory, as apoptosis resulting from mitochondrial membrane permeabilization is usually believed to be a caspase-dependent process. However, apoptosis can be mediated in caspase-independent manners as well. For instance, the release of the mitochondrial proteins Apotosis-inducing factor and endonuclease G have been shown to mediate programmed cell death in a caspase-independent manner after mitochondrial membrane permeabilization^39^,^40^. Moreover, Rege and colleagues showed that their KLAK conjugate can induce prostate cancer cell death through mitochondrial damage independently of caspase activation^14^. Nevertheless, our results clearly indicate that pHLIP-KLAKLAK toxicity results from disruption of the mitochondrial membrane potential in a pH-mediated manner.

Importantly, the low pH treatment has little to no effect on either MitoTracker Red and low Annexin V staining (Figs 5I and 6E, Bar Set #2). Interestingly and also consistent with the levels of LDH release and cytotoxicity observed when cells are treated with free peptides (Figs 4B,C and 5), (KLAKLAK)_2_ and KLAKLAK appear to induce PS externalization and loss of mitochondrial membrane potential in a pH-mediated manner, with (KLAKLAK)_2_ exhibiting the largest effect and KLAKLAK only a modest one (Fig. 6I, Bar Sets #6 vs #8). The effect of free KLAKLAK is significantly lower than with pHLIP-KLAKLAK when cells are treated at low pH. Noteworthy, there is no evidence of these effects 6 hours post-treatment with either constructs (Figure S3), which is consistent with the reported delayed action of the KLAK peptide on apoptosis upon its active translocation inside cells^10^,^15^.

Thus, all together, our results indicate that the anti-proliferative effect of pHLIP–KLAKLAK is due to the pH-selective translocation of KLAKLAK across the plasma membrane and to mitochondrial membrane disruption. It is crucial to note that pHLIP greatly potentiates the activity of KLAKLAK at lower pH, indicating a synergistic effect between pHLIP-mediated insertion and enhanced ‘permeability’ of KLAKLAK at low pH, which may contribute to the high activity of pHLIP-KLAKLAK and may be advantageous in animal settings.

## Conclusion

The use of AMPs have become more prevalent for the treatment of cancer but a lack of efficient and selective delivery system has limited their use and impact. Herein, we present a family of cell-impermeable (KLAKLAK)_2_ derivatives that exhibit a wide range of physical properties to determine the most efficacious therapeutic cargo peptide for targeting and translocation using pHLIP. Together, our results indicate that this family of (KLAKLAK)_2_ analogs can inhibit cancer cell growth in a concentration- and pH-dependent manner upon pHLIP-mediated translocation. Therefore, we anticipate that this approach could be applied to a broad range of therapeutic peptides with intracellular targets. In addition, we identified pHLIP-KLAKLAK as a lead conjugate because of its low cytotoxicity at physiological pH, chemical stability, high anti-proliferation potency and specific induction of mitochondrial membrane disruption at lower pH. Its translation to animal settings is the focus of current investigations.

## Methods

### Materials

*N*-Hydroxybenzotriazole (HOBt), *o*-benzotriazol-*N*, *N*, *N*, *N*′, *N*′-tetramethyluronium hexafluorophosphate (HBTU), and all *N*-fluorenyl-9-methoxycarbonyl (Fmoc) protected L-amino acids were purchased from GL Biochem Ltd. H-Rink Amide-ChemMatrix solid support resin was purchased from PCAS BioMatrix Inc., Diisopropylehtylamine (DIEA), Piperazine, *N*, *N*-dimethylformamide (DMF), dichloromethane (DCM), trifluoroacetic acid (TFA), methanol, acetonitrile, dimethyl sulfoxide (DMSO), and Dulbecco’s modified Eagle’s medium (DMEM) were all purchased from Thermo Fisher Scientific Inc. Fetal Bovine Serum (FBS) was purchased from Atlanta Biologicals Inc. Penicillin-Streptomycin was purchased from Sigma-Aldrich. 1-palmitoyl-2-oleoyl-sn-glycero-3-phosphocholine (POPC) was purchased from Avanti Polar Lipids Inc. 3-(4,5-dimethylthiazol-2-yl)-2,5-diphenyltetrazolium bromide (MTT) was purchased from EMD Millipore. Mitochondrial Membrane Potential/Annexin V Apoptosis Kit, Pierce LDH Cytotoxicity Assay Kit, and CellEvent™ Caspase-3/7 Green Detection Reagent were all purchased from Thermo Fisher Scientific.

### Solid-Phase Peptide Synthesis

pHLIP(WT) with a cysteine residue at its C terminus (GGEQNPIYWARYADWLFTTPLLLLDLALLVDADEGTCG) and KLAK peptides were prepared in our laboratory by Fmoc solid-phase synthesis using H-Rink Amide resin, affording an amidated C-terminus and a free N-terminal amine. Peptides were purified via reversed-phase high performance liquid chromatography (RP-HPLC) (Phenomenex Luna prep 10 μ 250 × 21.20 mm C8; flow rate 10 mL/min; phase A: water 0.1% TFA; phase B: acetonitrile 0.1% TFA; gradient 60 min from 95/5 A/B to 0/100 A/B. The purity of the peptides were determined by RP-HPLC as listed, and their identity was confirmed via matrix-assisted laser desorption ionization time of flight mass spectrometry (MALDI-TOF-MS): pHLIP(WT): purity >98%; calculated (M + H^+^) = 4212, found (M + H^+^) = 4211. CKLAKLAKKLAKLAK: purity >98%; calculated (M + H^+^) = 1627, found (M + H^+^) = 1627. CKLAKLAK: purity >98%; calculated (M + H^+^) = 874 (M + Na^2+^) = 896, found (M + Na^2+^) = 896. CKLAK: purity >98%; calculated (M + H^+^) = 562, found (M + H^+^) = 561. CKAAK: purity >98%; calculated (M + H^+^) = 520 (M + Na^2+^) = 542, found (M + Na^2+^) = 541. CKLLK: purity >98%; calculated (M + H^+^) = 604, found (M + H^+^) = 604. CKKKK: purity >98%; calculated (M + H^+^) = 634, found (M + H^+^) = 632. CKGGK: purity >98%; calculated (M + H^+^) = 492 (M + Na^2+^) = 514, found (M + Na^2+^) = 513.

### Preparation of pHLIP-KLAK Constructs

The pHLIP peptide was dissolved in DMSO to a concentration of 1 mM, followed by the addition of 1.5 eq. KLAK in DMSO and 50 μL of 1 M Tris buffer, pH 8.0 was added to the solution and allowed to mix at room temperature for 2–3 hours. The desired pHLIP conjugate was isolated using the same techniques described for the peptides. The purity of the peptide- conjugate was determined by RP-HPLC as listed, and their identity was confirmed by MALDI-TOF MS: pHLIP(WT)-(KLAKLAK)_2_: purity >98%; calculated (M + H^+^) = 5839, found (M + H^+^) = 5837. pHLIP(WT)-KLAKLAK: purity >98%; calculated (M + H^+^) = 5085, found (M + H^+^) = 5085. pHLIP(WT)-KLAK: purity >98%; calculated (M + H^+^) = 4773, found (M + H^+^) = 4775. pHLIP(WT)-KAAK: purity >98%; calculated (M + H^+^) = 4731, found (M + H^+^) = 4728. pHLIP(WT)-KLLK: purity >98%; calculated (M + H^+^) = 4815 (M + Na^2+^) = 4837, found (M + Na^2+^) = 4849. pHLIP(WT)-KKKK: purity >98%; calculated (M + H^+^) = 4845 (M + Na^2+^) = 4866, found (M + Na^2+^) = 4866. pHLIP(WT)-KGGK: purity >98%; calculated (M + H^+^) = 4703, found (M + H^+^) = 4710. The conjugates were quantified at 280 nm by UV/Vis absorbance spectroscopy using the molar extinction coefficient of pHLIP (13,940 M^−1^ cm^−1^) and lyophilized to 10^−8^ mole aliquots.

### Cell Culture

Human breast adenocarcinoma MDA-MB-231 (kind gift from Matthew Robinson, Fox Chase Cancer Center) were cultured in Dulbecco’s modified Eagle’s medium (DMEM supplemented with 10% FBS, 100 U/mL penicillin, and 0.1 mg/mL streptomycin in a humidified atmosphere of 5% CO_2_ at 37 °C.

### Anti-proliferation Assay

Cells were seeded in 96-well plates at a density of 3,000 cells/well and incubated overnight. Before treatment, construct aliquots were solubilized in an appropriate volume of DMEM without FBS (pH 7.4) so that upon pH adjustment the desired treatment concentration is obtained, and gently sonicated for 30–60 seconds using a bath sonicator (Branson Ultrasonics). After removal of cell media, this treatment solution was added to each well and incubated at 37 °C for 5–10 minutes. The pH was then adjusted to the desired pH using a pre-established volume of DMEM, pH 2.0 buffered with citric acid (final volume = 50 μL) and the plate was incubated at 37 °C for 2 hours. After treatment, the media was removed, cells were washed once with 100 μL of complete DMEM, and 100 μL of complete medium was added to each well before returning the plate to the incubator. Treatment solutions were collected and their pH values measured using a micro-combination pH probe (Microelectrodes, Inc.). For physiologic pH treatments, a small down-drift (~0.2 pH unit) was usually observed, whereas an up-drift of similar amplitude was observed for low pH treatments. Cell viability was determined after 72 hours using the colorimetric MTT assay. Briefly, 10 μL of a 5 mg/mL MTT stock solution was added to the treated cells and incubated for 2 hours at 37 °C. The resulting formazan crystals were solubilized in 200 μL DMSO and the absorbance measured at 580 nm using an Infinite 200 PRO microplate reader (Tecan). Cell viability was calculated against control cells treated with media at physiologic pH. When needed, data were fitted with a sigmoidal dose-response using Prism 5 for Macintosh (GraphPad, Inc).

### Cell Membrane Leakage Assay

For the Trypan Blue plasma membrane integrity assays cells were seeded in 96-well plates at a density of 3,000 cells/well and incubated until confluent (~72 hours). For the Lactate Dehydrogenase (LDH) assay cells were seed in 24-well plates at a density of 200,000 cells/well and incubated until confluent (~24 hours). Before treatments, construct aliquots were solubilized in an appropriate volume of DMEM without FBS (pH 7.4) so that upon pH adjustment 10 μM is obtained. Cell media was removed and the treatment solution was added to each well and incubated at 37 °C for 5–10 minutes. The pH was then adjusted to the desired pH using a pre-established volume of DMEM, pH 2.0 buffered with citric acid (final volume = 50 μL). The plate was incubated at 37 °C for 2 hours. After treatment, the media was removed, cells were detached using trypsin and counted based on trypan blue uptake using an hemacytometer. For the LDH assay cell media supernatant was collected in a 96 well plate in triplicate after treatment and LDH reaction mixture was added to each well recommended by the manufacturer protocol. After a 30 minute incubation at room temperature stop solution was added to each well and the absorbance at 490 and 680 nm of each well was determined using an Infinite 200 PRO microplate reader (Tecan). Percent LDH release was calculated against control cells treated with media at physiologic pH.

### Apoptosis Assays

Apoptosis was investigated by flow cytometry using the Mitochondrial Membrane Potential Apoptosis Kit with Alexa Fluor 488 Annexin V and MitoTracker Red (ThermoFisher Scientific). Cells were seeded in 24-well plates at a density of 200,000 cells/well and incubated until confluent (~24 hours). Before treatment, construct aliquots were solubilized in an appropriate volume of DMEM without FBS (pH 7.4) so that upon pH adjustment 10 μM is obtained. Cell media was removed and the treatment solution was added to each well and incubated at 37 °C for 5 minutes. The pH was then adjusted to the desired pH using a pre-established volume of DMEM, pH 2.0 buffered with citric acid (final volume 200 μL). The plate was incubated at 37 °C for 2 hours. After treatment, the media was removed, cells were washed once with 500 μL of complete DMEM, and recovered for 24 hours in complete DMEM. Cells were then pelleted, resuspended in 1 mL of complete DMEM and MitoTracker Red dye was added to the suspended cells following the manufacturer’s protocols and incubated for 30 min at 37 °C. The cells were then washed and incubated at room temperature with Alexa 488 Annexin V. The cells were then analyzed using a BDFacs Canto II flow cytometer (BD Biosciences, San Jose, CA) equipped with a 488 nm argon laser, and 530/633 bandpass filters. A minimum of 10,000 events were counted for each data point. Cells were gated as two populations (non-apoptotic and apoptotic cells) based on fluorescence intensity at both 530 nm and 633 nm wavelengths. The data was analyzed using the FACSDiva version 6.1.1 software.

#### Caspase Activity Assay

To probe the possible involvement of the caspase-mediated apoptotic pathway, we use the CellEvent™ Caspase-3/7 Green Detection Reagent (ThermoFisher Scientific). Cells were seeded at a density of 3,000 cells/well in a 96 well plate and allowed to adhere overnight. Before treatment, construct aliquots were solubilized in an appropriate volume of DMEM without FBS (pH 7.4) so that upon pH adjustment 10 μM is obtained. Cell media was removed and the treatment solution was added to each well and incubated at 37 °C for 5 minutes. The pH was then adjusted to the desired pH using a pre-established volume of DMEM, pH 2.0 buffered with citric acid (final volume 50 μL). The plate was incubated at 37 °C for 2 hours. After treatment, the media was removed, cells were washed once with 100 μL of complete DMEM, and recovered complete DMEM. As a positive control cells were also treated with 1 μM Staurosporine. Cells treated with pHLIP-KLAKLAK or free KLAK peptides were recovered and cells treated with Staurosporine were done so in the presence of CellEvent Caspase-3/7 Green Detection Reagent. Cells were monitored for the presence of active caspase at 24, 48 and 72 hours, based on the fluorescence intensity at excitation/emission at 485/530 nm using an Infinite 200 PRO microplate reader (Tecan). Caspase activity was normalized to control cells treated with media at physiologic pH.

## Additional Information

**How to cite this article**: Burns, K. E. *et al*. pH-Selective Cytotoxicity of pHLIP-Antimicrobial Peptide Conjugates. *Sci. Rep.* **6**, 28465; doi: 10.1038/srep28465 (2016).

## Supplementary Material

Supplementary Information

## Figures and Tables

**Table 1:** Properties of cargo peptides attached to the C-terminus of pHLIP.

C-terminal cargo	MW^a^	Log P_o/w_^a^
KGGK	490.6	−4.22
KAAK	518.7	−3.66
KLAK	560.8	−2.28
KLLK	602.8	−1.81
KKKK	632.9	−4.78
KLAKLAK	873.2	−3.49
(KLAKLAK)_2_	1625.1	−5.69

^a^Octanol/water partition coefficients (Log P_o/w_) and molecular weight (MW) were calculated using QikProp 3.0 (Schrödinger, LLC).
