# Supplementary material for: pH-Selective Cytotoxicity of pHLIP-Antimicrobial Peptide Conjugates
Source: Sci Rep. 2016 Jun 23;6:28465. doi: 10.1038/srep28465 (PMC4917822; doi:10.1038/srep28465)
Supplement: Supplementary Information [file srep28465-s1.pdf]

pH-Selective Cytotoxicity of pHLIP-Antimicrobial Peptide Conjugates

Kelly E. Burns, Tanner P. McCleerey and Damien Thévenin*
Department of Chemistry, Lehigh University, 6 East Packer Ave, Bethlehem PA 18015

*Corresponding author: damien.thevenin@lehigh.edu

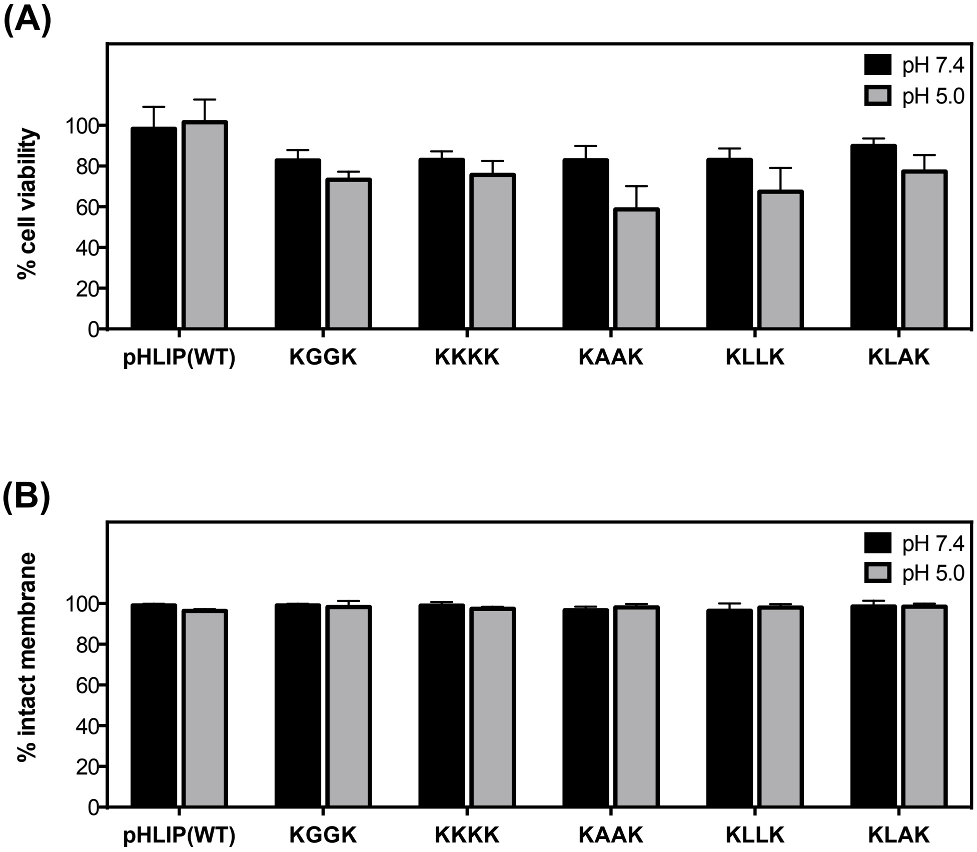

Figure S1. Effect of Free Peptides on Cell Viability and Plasma Membrane Integrity. For each condition, 3,000 cells/well (96-well plate) were seeded, incubated until confluent (~72 hours) and treated for 2 hours with 10 µM of free pHLIP(WT) or free tetrapeptides at pH 7.4 (black bars) and a low pH (grey bars). (A) Cell viability assay. After the 2-hour treatment, cells were washed once with media and cultured for 72 hour in complete medium at physiologic pH. Cell viability was assessed with the MTT assay. All measurements were normalized to the media control (0 µM, pH 7.4), as 100% cell viability. Results are shown as mean ± SEM (n=9-12). (B) After the 2-hour treatment, cells were detached and counted based on trypan blue uptake with an hemacytometer: % of intact cells corresponds to the number of cells not showing any dye uptake over the total number of cells. Results are shown as mean ± SD (n=3).

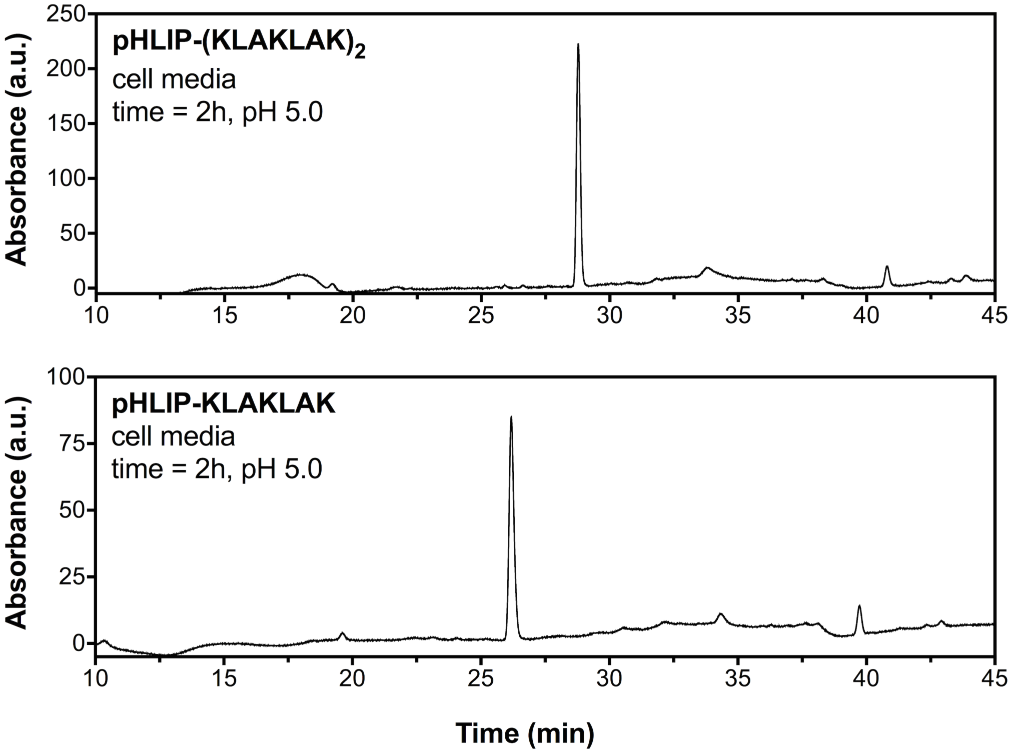

Figure S2. Chemical Stability of pHLIP conjugates. HPLC traces of 10 µM pHLIP-(KLAKLAK)$_2$ (A) and pHLIP-KLAKLAK (B) after incubation in cell media for 2h at pH 5.0 and 37 ºC. No significant degradation was observed for either of the pHLIP conjugates: For each HPLC run, every peak was collected and checked by mass spectroscopy. In all cases, the conjugates were found intact

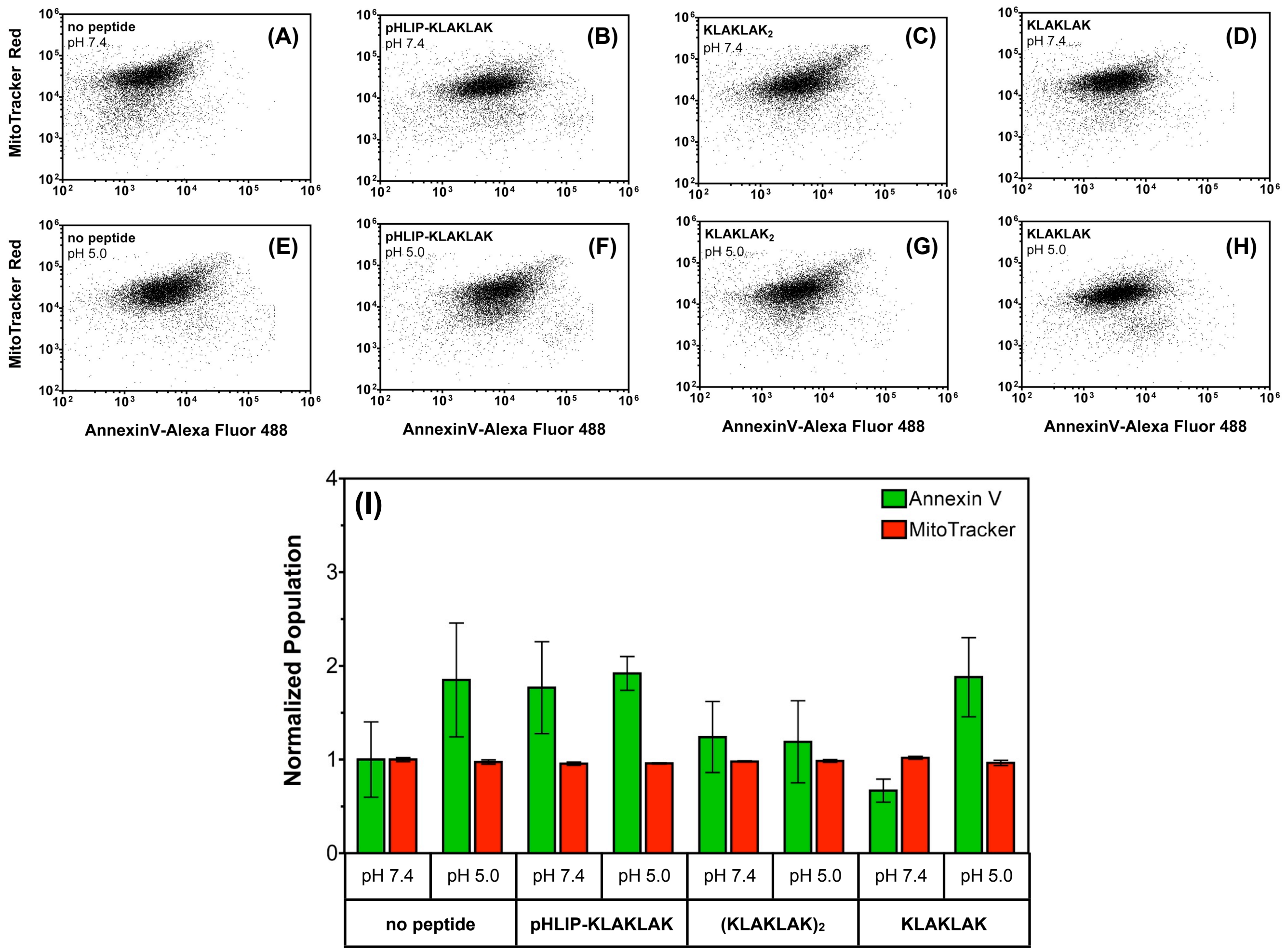

Figure S3. Peptides do not induce apoptosis within 6 hours post-treatment. Annexin V - AlexaFluor 488 and MitoTracker Red dual staining was performed and measured by flow cytometry to evaluate the induction of apoptosis in MDA-MB-231 cells by pHLIP-KLAKLAK, $(KLAKLAK)_2$ and KLAKLAK. (A-H) Cells were treated with either no peptide or with 10 µM peptides at pH 7.4 (A-D) or at pH 5.0 (E-H) for 2 hours, washed once with media and cultured for 6 hours in complete medium at physiologic pH. The relative amount of cells in each population is shown in (I). A minimum of 10,000 events were counted for each data point. The data was analyzed using the FACSDiva version 6.1.1 software. Results are shown as mean ± SD (n=3). Multiple comparisons analyses using either the Dunnett (at 95% confidence intervals) or the Holm-Ŝidák tests (Prism 6 for Macintosh) show no statistically significant differences between the treatment conditions and the pH 7.4 no peptide control.

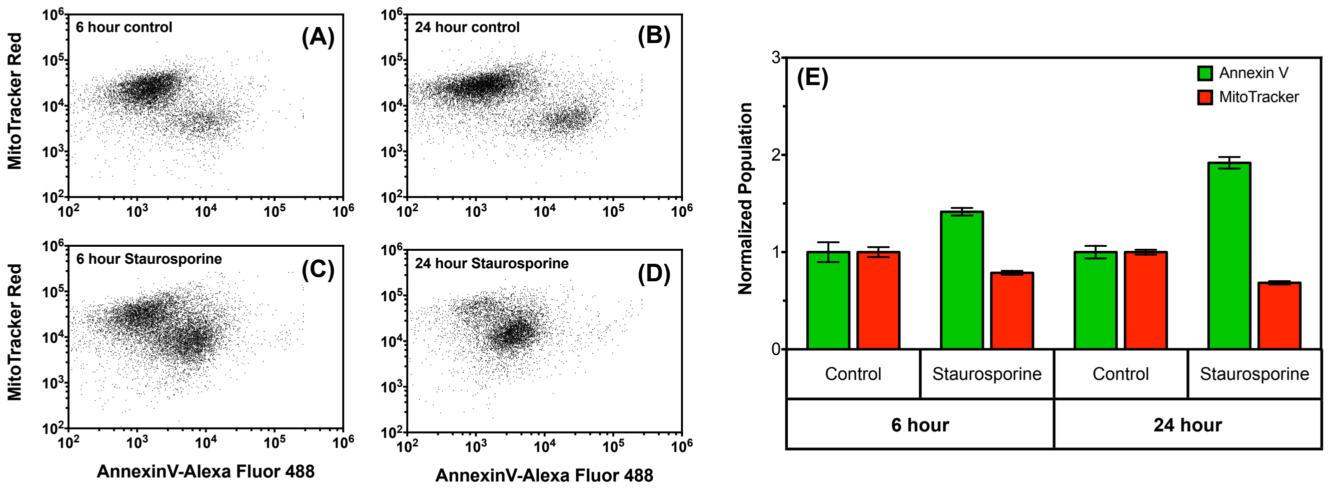

Figure S4. Staurosporine Apoptosis Positive Control. Annexin V -AlexaFluor 488 and MitoTracker Red dual staining was performed and measured by flow cytometry to evaluate the induction of apoptosis in MDA-MB-231 cells by Staurosporine. Cells were treated with either no compound (A-B) or with 1 μM Staurosporine (C-D) for either 6 (A and C) or 24 hours (B and D). The relative amount of cells in each population is shown in (E). A minimum of 6,000-10,000 events were counted for each data point. The data was analyzed using the FACSDiva version 6.1.1 software. Results are shown as mean ± SD (n=3).

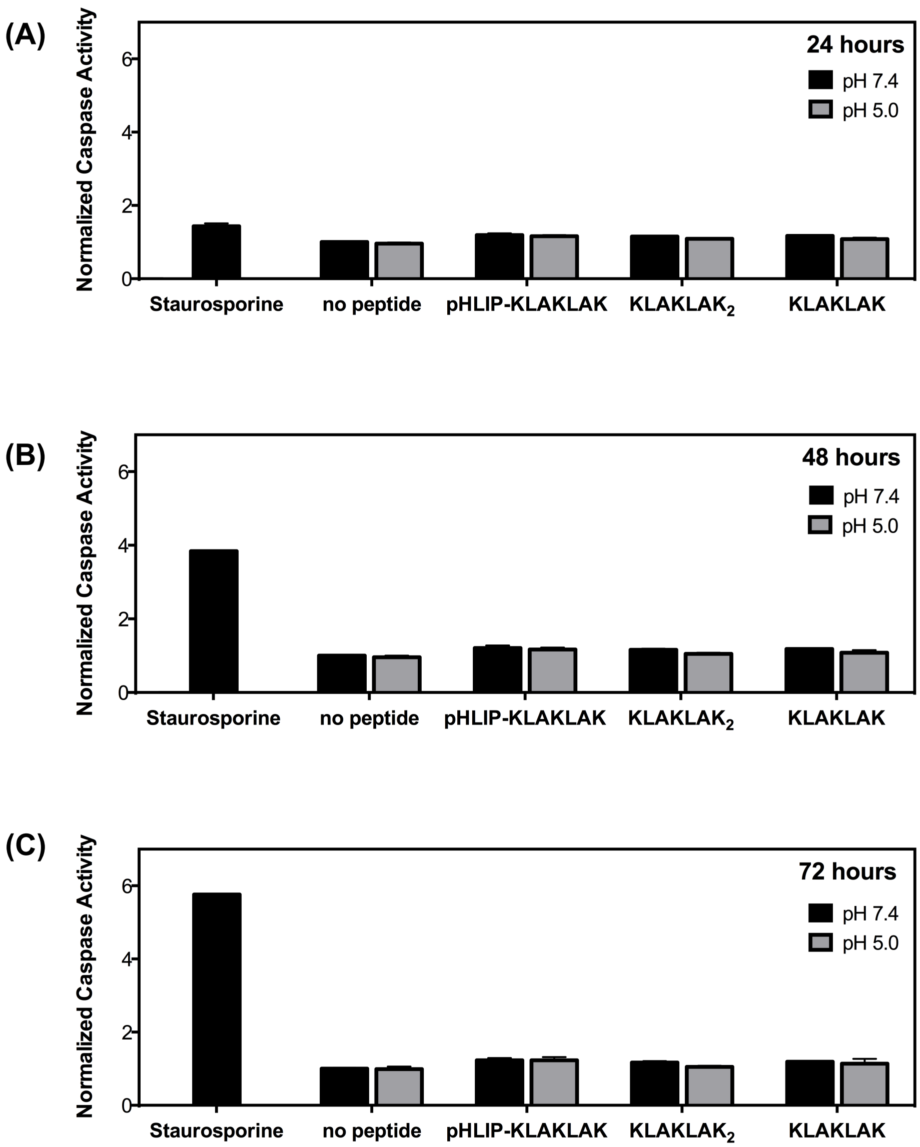

Figure S5. Caspase Activity Assay. Cells were seeded at a density of 3,000 cells/well in a 96 well plate, allowed to adhere overnight and treated with either 10 μM peptides for 2 hours at pH 7.4 (black bars) or pH 5.0 (grey bars) or 1 μM Staurosporine. Cells treated with pHLIP-KLAKLAK or free KLAK peptides were recovered in complete medium and cells treated with Staurosporine were treated in the presence of CellEvent Caspase-3/7 Green Detection Reagent. Cells were monitored for the presence of active caspase at 24, 48 and 72 hours, A-C respectively. Results are shown as mean ± SD (n=3).
